# Characteristics of multidrug-resistant *Acinetobacter baumannii* strains isolated in Geneva during colonization or infection

**DOI:** 10.1186/s12941-015-0103-3

**Published:** 2015-09-11

**Authors:** Abdessalam Cherkaoui, Stéphane Emonet, Gesuele Renzi, Jacques Schrenzel

**Affiliations:** Bacteriology Lab, Division of Laboratory Medicine, Department of Genetics and Laboratory Medicine, Geneva University Hospitals, 4 rue Gabrielle-Perret-Gentil, 1205 Geneva, Switzerland; Genomic Research Laboratory, Service of Infectious Diseases, Geneva University Hospitals, 4 rue Gabrielle-Perret-Gentil, 1205 Geneva, Switzerland

**Keywords:** Multi-drug resistant, *Acinetobacter baumannii*, β-lactamase genes, PCR, DiversiLab, rep-PCR

## Abstract

This study determined the antibiotic susceptibility profile and genetic mechanisms of β-lactam resistance in 27 clinical strains of *Acinetobacter baumannii* isolated at the University Hospitals of Geneva, Switzerland. The antimicrobial susceptibility testing was performed using Etest and the disc diffusion method in accordance with CLSI guidelines. All of the strains were defined as multi-drug resistant (MDR) and were susceptible to colistin and moderately susceptible to tigecycline. Uniplex PCR assays were used to detect the following β-lactamase genes: four class D carbapenem-hydrolysing oxacillinases (*bla*OXA-51, *bla*OXA-23, *bla*OXA-24 and *bla*OXA-58), four class B metallo-β-lactamases genes (*bla*IMP, *bla*VIM, *bla*SPM and *bla*NDM) and two class A carbapenemases (*bla*KPC and *bla*GES). All of the strains were positive for *bla*OXA-51 (intrinsic resistance), 14/27 strains carried *bla*OXA-23, 2/27 strains carried a *bla*OXA-24-like gene, and 4/27 strains had a *bla*OXA-58 gene. *bla*GES-11 was found in three strains, and NDM-1-harbouring strains were identified in three patients. All of the *A. baumannii* isolates were typed by rep-PCR (DiversiLab) and excluded any clonality. Altogether, this analysis suggests a very high genetic diversity of imported MDR *A. baumannii*.

## Background

*Acinetobacter baumannii*, a Gram-negative opportunistic coccobacilli, has emerged globally in healthcare institutions because it is hard to eradicate, most likely because it is resistant to desiccation and to ultraviolet and chemical sanitizers [1]. *A. baumannii* displays numerous intrinsic and acquired drug-resistance mechanisms. Of the multidrug-resistant organisms, the highly resistant *Acinetobacter* spp. isolates deserve special mention. These organisms can be resistant to all of the currently available antimicrobial agents or remain susceptible only to older, potentially more toxic agents, such as polymyxins, leaving limited and suboptimal options for treatment [2]. Carbapenem- and colistin-resistant *A.* *baumannii* infections were recently reported in two Sicilian hospitals [3]. *Acinetobacter* spp. may develop resistance to carbapenems through various mechanisms, including class B and D carbapenemase production, decreased permeability, altered penicillin-binding proteins, and even in some cases the overexpression of efflux pumps [4, 5]. Carbapenem resistance in *Acinetobacter* species is most commonly caused by the production of OXA-type carbapenemases and metallo-β-lactamases (MBLs) [6, 7]. The OXA-type carbapenemases comprise four broad groups: *bla*OXA-23-like, *bla*OXA-40-like, *bla*OXA-58-like and an intrinsic *bla*OXA-51-like [8–10]. The OXA-51-like β-lactamases are intrinsic to *A. baumannii* and have therefore been used as a method of species identification [8, 11]. The MBLs require a zinc ion for their activity, which is inhibited by metal chelators, such as EDTA, and thiol-based compounds but not by sulbactam, tazobactam or clavulanic acid. Among the multiple types of MBL genes described throughout the world, the *bla*IMP and *bla*VIM types are the most common [9]. The genes responsible for MBL production may be chromosomal or plasmidic, and the latter pose a threat of horizontal transfer among Gram-negative bacteria [12]. To date, infections associated with *A. baumannii* New Delhi metallo-β-lactamase-1 (NDM-1)-positive strains have been reported in several countries, including Switzerland [13, 14]. The genes encoding NDM-1 are carried on a plasmid, thus promoting the spread of resistance among Gram-negative organisms, most likely by horizontal gene transfer [15, 16]. An NDM-2 variant (Pro-to-Ala substitution at position 28) was recently described [17]. This allele was first found in a multidrug-resistant *A. baumannii* strain isolated from a German patient previously hospitalised in Egypt and subsequently isolated in Israel [12, 17, 18].

Despite the implementation of infection control measures, *A. baumannii* remains an important problem in many healthcare institutions around the world. Several studies have reported high rates of faecal carriage of *Acinetobacter*, making the digestive tract a potential reservoir for nosocomial infections and outbreaks caused by multidrug-resistant *Acinetobacter* strains [19, 20]. To control the dissemination of these organisms, rectal swabs are routinely collected from admitted patients based on their recent travel history or hospitalization in foreign countries. The infection control policies that are implemented at the institution where this study was performed request the screening of admitted patients based on their recent travel history or hospitalization in foreign countries. Rectal swab specimens (e-Swab, Copan, Brescia, Italy) from patients fulfilling these criteria were screened for multi-resistant *A.* *baumannii* using CHROMagar ESBL (Becton, Dickinson, Allschwil, Switzerland) and MacConkey plates (bioMérieux, Geneva, Switzerland) with imipenem, meropenem, and ertapenem disks (MacD) [21]. Suspected colonies were sub-cultured on sheep blood agar for identification (MALDI Biotyper 3.0, Bruker Daltonics, Bremen, Germany) and full antimicrobial susceptibility testing according to CLSI criteria. Multidrug resistance is defined as resistance to at least two different classes of antibiotics [22].

The aim of the present study was to investigate the β-lactam resistance mechanisms involved in a collection of multidrug-resistant *A. baumannii* strains isolated at Geneva University Hospitals, Switzerland.

## Methods

### Strains and growth conditions

The study included 27 non-duplicate multidrug-resistant *A. baumannii* strains recovered from clinical specimens. Most of the isolates were obtained from rectal swabs of colonised patients during routine screening, whereas a minority of the clinical specimens comprised samples of urine, exudative skin specimens, and lower respiratory tract secretions (Table 1). All of the strains were stored at −80 °C in skim milk with 15 % glycerol. The *A. baumannii* strains were grown on Columbia blood agar at 37 °C with 5 % CO_2_, and their identification was confirmed by MALDI-TOF/MS [23].

**Table 1:** Antimicrobial susceptibility of multidrug-resistant *Acinetobacter baumannii* isolates collected at Geneva University, Switzerland (n = 27)

Strain ID	Age, years	Gender	Origin	Sample type	E-test / MIC (mg/L)				Carbapenemase				
					IMI	MER	TIG	COL	NDM-1	GES-11	OXA-23	OXA-24	OXA-58
1	66	male	Switzerland	skin swab	128	64	6	0.19			**+**		
2	50	male	Pakistan	rectal swab	48	48	3	0.094			**+**		
3	35	male	Libya	rectal swab	64	48	4	0.19		**+**	**+**		
4	76	male	Switzerland	urine	64	64	1.5	0.094			**+**		
5	66	male	Switzerland	rectal swab	64	48	4	0.19	**+**		**+**		
6	40	female	Sudan	rectal swab	128	64	3	0.19				**+**	
7	72	male	Switzerland	rectal swab	192	64	3	0.064			**+**		
8	2	male	ND	rectal swab	64	64	6	0.19			**+**		
9	78	male	Thailand	wound swab	128	192	3	0.125			**+**		
10	72	male	ND	urine	32	12	3	0.094					**+**
11	68	male	Switzerland	rectal swab	64	192	6	0.25					
12	20	male	Syria	rectal swab	128	256	2	0.19				**+**	
13	79	male	Switzerland	rectal swab	192	192	4	0.19			**+**		
14	27	female	Egypt	rectal swab	64	48	3	0.19			**+**		
15	62	male	Roumania	rectal swab	64	48	3	0.19					**+**
16	35	male	Libya	blood	48	64	16	0.19			**+**		
17	27	male	Switzerland	respiratory sample	24	3	4	0.19					**+**
18	41	male	Kosovo	skin swab	192	24	3	0.19					**+**
19	80	male	Switzerland	wound swab	64	192	3	0.094			**+**		
20	ND	male	Libya	wound swab	12	64	1	0.25					
21	64	male	Libya	rectal swab	12	24	0.25	0.094		**+**			
22	77	female	Switzerland	rectal swab	192	192	3	0.38					
23	62	male	Switzerland	wound swab	256	256	3	0.25					
24	48	male	Switzerland	rectal swab	256	256	4	0.25			**+**		
25	71	male	England	skin swab	6	32	4	0.25		**+**			
26	43	male	Egypt	rectal swab	24	32	3	0.5	**+**		**+**		
27	24	male	Egypt	rectal swab	256	64	4	0.38	**+**				
				**MIC 50**	64	64	3	0.19					
				**MIC 90**	192	192	6	0.25					

ND, not determined; IMI, imipenem; MER, meropenem; TIG, tigecycline; COL, colistin

### Antimicrobial susceptibility testing

The susceptibility to various classes of antibiotics was determined by Etest (bioMérieux SA, Geneva, Switzerland) and the disc diffusion method in accordance with CLSI guidelines. The antibiotics tested were amikacin (30 μg), ciprofloxacin (5 μg), ceftazidime (30 μg) and piperacillin-tazobactam (100/10 μg). The minimum inhibitory concentrations (MICs) of imipenem, meropenem, tigecycline, and colistin were determined using the Etest method. The interpretation breakpoints were based on published data from the Clinical Laboratory Standards Institute (CLSI). The colistin and tigecycline MICs were interpreted using the European Clinical Antimicrobial Susceptibility Testing (EUCAST) guidelines (*Acinetobacter* breakpoints for colistin and *Enterobacteriaceae* breakpoints for tigecycline).

### Detection of carbapenem resistance genes by polymerase chain reaction (PCR)

The DNA from an overnight culture on Columbia blood agar was extracted using a MagNA Pure LC instrument (Roche, Rotkreuz, Switzerland) according to the manufacturer’s instructions. The final elution volume was 100 μl, and 5 µl of the DNA extract was used for each PCR analysis. Uniplex PCR assays were used to detect the following β-lactamase genes: four carbapenem-hydrolysing oxacillinases (*bla*OXA-51, *bla*OXA-23, *bla*OXA-24 and *bla*OXA-58), four metallo-β-lactamases genes (*bla*IMP, *bla*VIM, *bla*SPM and *bla*NDM), *bla*KPC, and *bla*GES. The primers used for PCR amplification of the carbapenemase genes are listed in Table 2.

**Table 2:** Primers used in the amplification of selected carbapenemase genes

Name	Nucleotide sequence (5′ → 3′)	Product size (bp)	Location	References
OXA-23-like	F- GATCGGATTGGAGAACCAGA	501	*bla*OXA-23	[31]
	R- ATTTCTGACCGCATTTCCAT			
OXA-24-like	F- GGTTAGTTGGCCCCCTTAAA	246	*bla*OXA-24	[31]
	R- AGTTGAGCGAAAAGGGGATT			
OXA-51-like	F- TAATGCTTTGATCGGCCTTG	353	*bla*OXA-51	[31]
	R- TGGATTGCACTTCATCTTGG			
OXA-58-like	F- AAGTATTGGGGCTTGTGCTG	599	*bla*OXA-58	[31]
	R- CCCCTCTGCGCTCTACATAC			
IMP^a^	F- GGAATAGAGTGGCTTAA**Y**TCTC	232	*bla*IMP	[32]
	R- GGTTTAA**Y**AAAACAACCACC			
VIM	F- GATGGTGTTTGGTCGCATA	390	*bla*VIM	[32]
	R- CGAATGCGCAGCACCAG			
SPM	F- AAAATCTGGGTACGCAAACG	271	*bla*SPM	[32]
	R- ACATTATCCGCTGGAACAGG			
NDM	F- GGTTTGGCGATCTGGTTTTC	621	*bla*NDM	[32]
	R- CGGAATGGCTCATCACGATC			
GES	F-ATGCGCTTCATTCACGCAC	863	*bla*GES	[33]
	R-CTATTTGTCCGTGCTCAGGA			
GES	F- CGGTTTCTAGCATCGGGACACAT	263	*bla*GES	[34]
	R- CCGCCATAGAGGACTTTAGCMACAG			
	*Probe*: Quasar 705-CGACCTCAGAGATACAACTACGCCTATTGC-BHQ2			
NDM-1	F- ATTAGCCGCTGCATTGAT	154	*bla*NDM	[35]
	R- CATGTCGAGATAGGAAGTG			
	***Probe***: FAM-CTG[+ C]CA [+ G]AC [+ A]TT [+ C]GG TGC-TAMRA			
KPC	F- GATACCACGTTCCGTCTGG	246	*bla*KPC	[36]
	R- GCAGGTTCCGGTTTTGTCTC			
	***Probe***: FAM-AGCGGCAGCAGTTTGTTGATTG-3′–BHQ1			

^a^Y = C or T

### Molecular typing methods

The DNA was extracted as described above and amplified using the DiversiLab *Acinetobacter* kit (bioMérieux, La Balme-les-Grottes, France) for DNA fingerprinting according to the manufacturer’s instructions. PCR was run on a preheated thermal cycler using the parameters recommended by the manufacturer. The kit-specific positive and negative controls were run with each reaction set to validate the amplification. The rep-PCR products were detected, and the amplicons were separated using microfluidics lab-on-a-chip technology and analysed using the DiversiLab system. Further analysis was performed with the web-based DiversiLab software (version 3.4) using the band-based modified Kullback–Leibler distance for the calculation of the percent similarities. The manufacturer provides guidelines for strain-level discrimination: strains with greater than 97 % similarity are considered indistinguishable (no differences in fingerprints), strains with greater than 95 % similarity are considered similar (1- to 2-band differences in fingerprints), and strains with less than 95 % similarity are considered different. In this study, the optimal cut-off for clustering was 95 %.

## Results and discussion

### Antimicrobial susceptibility

All of the isolates analysed in this study were resistant to amikacin, ciprofloxacin, ceftazidime, and piperacillin-tazobactam. The MIC of imipenem ranged from 6 to 256 mg/L, and the MIC of meropenem ranged from 3 to 256 mg/L. The MIC50 and MIC90 values for imipenem and meropenem were 64 and 192 mg/L, respectively. All of the tested isolates were susceptible to colistin. The MIC of colistin ranged from 0.064 to 0.5 mg/L, and the MIC50 and MIC90 values for colistin were 0.19 and 0.25 mg/L, respectively. The MIC of tigecycline ranged from 0.25 to 16 mg/L, whereas the MIC50 and MIC90 values for tigecycline were 3 and 6 mg/L, respectively. Susceptibility to tigecycline was observed in two (7.4 %) of the 27 *A. baumannii* isolates.

### Bacterial isolates, species identification and molecular analysis

Carbapenem resistance in *A. baumannii* is most often associated with class D β-lactamases (OXA-23-like, OXA-40-like and OXA-58-like) and MBLs. OXA-type carbapenemases are predominant in *A. baumannii*, particularly in worldwide outbreaks of OXA-23 [24]. The molecular analysis of the isolates tested in this study revealed that 14 strains (51.8 %) carried the *bla*OXA-23-like gene and that two strains carried a *bla*OXA-24-like gene. All of the strains had a *bla*OXA-51-like gene, and four strains had a *bla*OXA-58 gene. In this study, the OXA-58 isolates presented lower MIC values for meropenem than OXA-23-like-positive isolates, which systematically exhibited higher MIC values (Table 1). The isolates with non-acquired OXA genes displayed a marked variation and included some carbapenem-resistant genes. Naturally occurring OXA carbapenemases, such as OXA-51-like enzymes (e.g., OXA 64-66, OXA 68-71, OXA 78-80, OXA-82, OXA-86, OXA-92 and OXA104-112), have been identified in *A. baumannii* isolates worldwide. In addition, strains producing OXA-58 derivatives have been found in isolates recovered from Italy, Belgium, France, Greece, Iran, the United States and Argentina [25]. OXA-23, OXA-58 and OXA-51 have been reported in Turkey [25, 26]. All of the strains investigated in this study had an intrinsic blaOXA-51-like gene, supporting the use of this resistance gene as a surrogate for the identification of a strain as *A.* *baumannii* [27]. Recently, the presence of Ambler class A GES (Guiana extended-spectrum) β-lactamases has also been reported in *A.* *baumannii* and can confer a high level of resistance to carbapenems [8]. *bla*GES genes have been reported in several countries in Europe, Asia, South America and South Africa [8]. In addition, several GES-1 mutants, such as GES-11, GES-12, GES-14, and GES-22, have been detected in *A. baumannii* [8]. In the present study, *bla*GES-11 was detected in three strains, one of which also carried a *bla*OXA-23-like gene (Table 1). The most important characteristic of the GES family of enzymes is their ability to evolve into carbapenemases. GES and OXA-type enzymes are jointly responsible for the high carbapenem-resistance levels observed in the tested strains. The emergence of NDM-1-producing *Acinetobacter* spp. has been recently reported in many countries, such as India, Israel, Egypt, Germany, Spain, Switzerland, the United Arab Emirates and China [13, 28, 29]. Interestingly, *bla*NDM-1 has been shown to be a chimeric gene constructed by the fusion of the aminoglycoside-resistance gene *aphA6* with a mannose-binding lectin (MBL) gene, an event that most likely occurred in *Acinetobacter* spp., indicating that *Acinetobacter* spp. are the likely origin of this gene [30]. *A. baumannii* is the most common NDM-1-producing *Acinetobacter* spp., and the *bla*NDM-1 gene is mostly chromosomal [13, 16]. In this study, NDM-1-harbouring strains were identified in three patients, and two of these originated from Egypt. These isolates have been found to carry mixed carbapenemase genes (*bla*OXA-23 with *bla*NDM-1), yielding very broad-spectrum antibiotic resistance profiles. These isolates are susceptible only to colistin. Taking into account the relationship between North African countries and many European countries, it is possible that the spread of NDM-1 carbapenemases may occur rapidly, mostly through *A. baumannii* rather than *Enterobacteriaceae* because *A. baumannii* may become markedly more difficult to eradicate [28]. *bla*KPC was not detected in any of the 27 isolates. The emergence and coexistence of these major resistance mechanisms seriously limit therapeutic options, raising concerns regarding their transmission to other organisms. It is important to highlight the fact that most of the multidrug-resistant isolates analysed in this study were derived from intestinal colonization and that this—mostly unrecognized—carriage in hospitalized patients may constitute a reservoir of *A. baumannii* strains that are not susceptible to carbapenem.

**Fig. 1:**
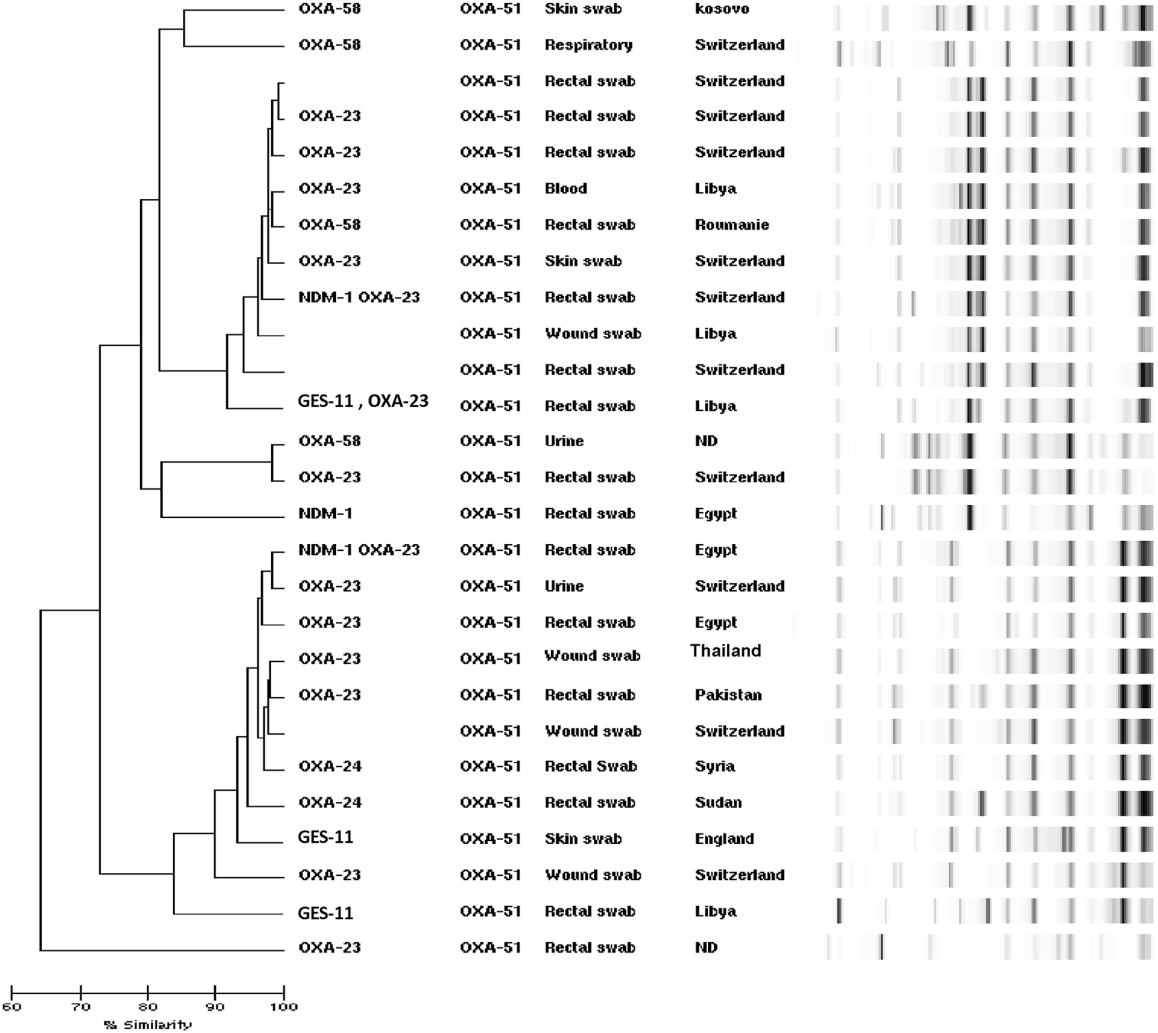
Results of the DiversiLab typing analysis of isolates from patients infected/colonised with multidrug-resistant *Acinetobacter baumannii* strains from Geneva University Hospitals, Switzerland (n = 27)

The genomic pattern of all of the isolates revealed a high degree of variability. Neither a dendrogram nor a computer-generated image of rep-PCR banding patterns showed clustering between the three oxacillinase genes (OXA-23-like, OXA-24-like and OXA-58; Fig. 1).

## Conclusion

In recent years, *A. baumannii* has emerged as one of the most challenging pathogens responsible for healthcare-associated infections. Despite being less frequently identified in *A. baumannii* than OXA-type carbapenemases, MBLs exhibit significantly more potent hydrolytic activities against carbapenems. Interestingly, the results of this study provide evidence that NDM-encoding genes may be widespread in *A. baumannii* and that further molecular surveys will be necessary to more carefully evaluate their distribution in this species. The routine implementation of simple and inexpensive screening methods to detect carbapenemase production in microbiology laboratories is therefore crucial for the optimal treatment of patients infected with carbapenemase-producing pathogens and for controlling the spread of resistance.
